# CoSFISH: a comprehensive reference database of COI and 18S rRNA barcodes for fish

**DOI:** 10.1093/database/baae038

**Published:** 2024-05-27

**Authors:** Yuanyuan Wang, Yexin Yang, Yi Liu, Chao Liu, Meng Xu, Miao Fang, Xidong Mu

**Affiliations:** Key Laboratory of Prevention and Control for Aquatic Invasive Alien Species, Ministry of Agriculture and Rural Affairs, Guangdong Modern Recreational Fisheries Engineering Technology Center, Pearl River Fisheries Research Institute, Chinese Academy of Fishery Sciences, No.1 Xingyu Road, Xilang, Liwan District, Guangzhou 510380, China; Key Laboratory of Prevention and Control for Aquatic Invasive Alien Species, Ministry of Agriculture and Rural Affairs, Guangdong Modern Recreational Fisheries Engineering Technology Center, Pearl River Fisheries Research Institute, Chinese Academy of Fishery Sciences, No.1 Xingyu Road, Xilang, Liwan District, Guangzhou 510380, China; Guangdong Provincial Key Laboratory of Aquatic Animal Immunology and Sustainable Aquaculture, No.1 Xingyu Road, Xilang, Liwan District, Guangzhou 510380, China; Key Laboratory of Prevention and Control for Aquatic Invasive Alien Species, Ministry of Agriculture and Rural Affairs, Guangdong Modern Recreational Fisheries Engineering Technology Center, Pearl River Fisheries Research Institute, Chinese Academy of Fishery Sciences, No.1 Xingyu Road, Xilang, Liwan District, Guangzhou 510380, China; Key Laboratory of Prevention and Control for Aquatic Invasive Alien Species, Ministry of Agriculture and Rural Affairs, Guangdong Modern Recreational Fisheries Engineering Technology Center, Pearl River Fisheries Research Institute, Chinese Academy of Fishery Sciences, No.1 Xingyu Road, Xilang, Liwan District, Guangzhou 510380, China; Key Laboratory of Prevention and Control for Aquatic Invasive Alien Species, Ministry of Agriculture and Rural Affairs, Guangdong Modern Recreational Fisheries Engineering Technology Center, Pearl River Fisheries Research Institute, Chinese Academy of Fishery Sciences, No.1 Xingyu Road, Xilang, Liwan District, Guangzhou 510380, China; Key Laboratory of Prevention and Control for Aquatic Invasive Alien Species, Ministry of Agriculture and Rural Affairs, Guangdong Modern Recreational Fisheries Engineering Technology Center, Pearl River Fisheries Research Institute, Chinese Academy of Fishery Sciences, No.1 Xingyu Road, Xilang, Liwan District, Guangzhou 510380, China; Key Laboratory of Prevention and Control for Aquatic Invasive Alien Species, Ministry of Agriculture and Rural Affairs, Guangdong Modern Recreational Fisheries Engineering Technology Center, Pearl River Fisheries Research Institute, Chinese Academy of Fishery Sciences, No.1 Xingyu Road, Xilang, Liwan District, Guangzhou 510380, China

## Abstract

Fish, being a crucial component of aquatic ecosystems, holds significant importance from both economic and ecological perspectives. However, the identification of fish at the species level remains challenging, and there is a lack of a taxonomically complete and comprehensive reference sequence database for fish. Therefore, we developed CoSFISH, an online fish database. Currently, the database contains 21 535 cytochrome oxidase I sequences and 1074 18S rRNA sequences of 21 589 species, belonging to 8 classes and 90 orders. We additionally incorporate online analysis tools to aid users in comparing, aligning and analyzing sequences, as well as designing primers. Users can upload their own data for analysis, in addition to using the data stored in the database directly. CoSFISH offers an extensive fish database and incorporates online analysis tools, making it a valuable resource for the study of fish diversity, phylogenetics and biological evolution.

**Database URL:**  http://210.22.121.250:8888/CoSFISH/home/indexPage.

## Introduction

Fish comprises over 50% of the total number of vertebrates globally, with an estimated 60 000 species (1), of which more than 35 600 species have been recorded (2). Due to their economic and ecological significance, fish have attracted a lot of attention, especially economic fish in the aquaculture industry and model fish utilized in scientific research. Accurate identification of fish species is essential for any fish-based studies. However, species-level classification of fish remains challenging. Morphological features can sometimes cause confusion in identification, as individuals of the same species exhibit morphological flexibility, including fish (3). Therefore, molecular markers have been developed to fill this gap. Among the wide variety of molecular markers, mitochondrial DNA markers are widely used in species delineation and phylogenetic analysis, due to their maternal inheritance, non-recombination and higher mutation rate than nuclear DNA markers (4). Among mitochondrial DNA markers, mitochondrial cytochrome oxidase I (COI) is most widely used for DNA barcoding (5). However, the resolution of mitochondrial DNA markers can be limited, particularly due to the common occurrence of fish hybrids, making them insufficient for species classification alone. Therefore, nuclear markers are essential for precise species identification and population genetic analyses. Combining mitochondrial molecular markers, such as Cytb, COI and 16S rRNA (5), and nuclear molecular markers, such as ITS1, ITS2, 18S rRNA and 28S rRNA (6), provides more comprehensive genetic insights. This enables greater information richness, broader field application and a deeper understanding of genetic diversity and evolution.

For fish, several databases have been developed, such as FishBase, Mitofish, FishDB, Fish-T1K, FishGET, FishSCT, FishSED and cnfishbase. FishBase (https://www.fishbase.se/home.php) is a comprehensive online database dedicated to global fish species, offering detailed information covering taxonomy, distribution, morphology, ecology, behavior and biology, accompanied by corresponding pictures. Mitofish (http://mitofish.aori.u-tokyo.ac.jp) is a database particularly for fish mitogenomes and provides online mitogenome assembly, annotation and phylogenetic analysis as well (7, 8). FishDB (http://fishdb.ihb.ac.cn) integrates fish genomes and transcriptomes and allows online comparative genomics analysis on orthologs (9). Fish-T1K (https://db.cngb.org/fisht1k), FishGET (http://bioinfo.ihb.ac.cn/fishget), FishSCT (http://bioinfo.ihb.ac.cn/fishsct) and FishSED (http://bioinfo.ihb.ac.cn/fishsed) are all specific for fish transcriptomes, enhancing our understanding of fish biology and evolution (10–13). cnfishbase (https://cnfishbase.cn) specially collects data of Chinese fishes, including their taxonomy and distribution visualization (14). However, the creation of a taxonomically exhaustive and comprehensive reference sequence database for fish is of utmost importance to facilitate the widespread implementation of DNA barcoding and metabarcoding methodologies in the monitoring and evaluation of aquatic ecosystems. Compiling regional data that facilitate large-scale fish diversity research continues to pose a significant challenge. Furthermore, the identification of fish species, especially hybrids, is currently difficult. In this study, we therefore developed the CoSFISH database, including COI and 18S rRNA sequences for recorded global fish species, to provide a more comprehensive view of fish diversity and phylogenetics.

## Materials and methods

### Data collection

As COI is the most extensively utilized mitochondrial DNA marker for fish, we incorporated COI barcodes within the CoSFISH database. We examined various nuclear markers for fish, such as 5.8S, ITS1, ITS2, 18S and 28S, and discovered that 18S rRNA was the most common marker among the fish species. As a result, our database specializes in using COI and 18S rRNA as the principle mitochondrial and nuclear markers, respectively. According to the taxonomic level of fish species in the book *Fishes of the World* (fifth edition) (1), we collected basic fish species information, such as species names, pictures, taxonomy and distribution information, from the FishBase website (https://www.fishbase.se/home.php) (2) and downloaded their COI and 18S rRNA sequences from NCBI (https://www.ncbi.nlm.nih.gov/) (15) correspondingly. The longest sequence was selected when one species possessed more than one COI/18S rRNA sequence in NCBI. However, it is worth noting that NCBI lacks precise taxonomic-level information for some sequences. Therefore, we cross-referenced these sequences with the bold database (http://www.barcodinglife.org) (16) to finalize the dataset.

### Database construction

The CoSFISH database was deployed on the Ubuntu 20.04 operation system and developed using AKKA 2.13 (web server), MySQL 8.0.30 (database server), Scala 2.13.2 and SBT 1.3.9. All data in the database were managed and stored using the MySQL Database Management System. The query function was enforced based on Slick 3.3.2 middleware tier. The website interface components were designed and implemented using Bootstrap 4.6.0 and Play Framework 2.8.7. The website has been tested in multiple popular web browsers, including Firefox, Google Chrome and Internet Explorer, and is publicly available at http://210.22.121.250:8888/CoSFISH/home/indexPage.

## Results

### CoSFISH data content

A total of 21 589 fish species, belonging to 8 classes and 90 orders, were included in this study. A total of 21 535 COI sequences and 1074 18S rRNA sequences were mined from NCBI, respectively. Additionally, their species names, pictures, taxonomy and distribution information were correspondingly included based on FishBase. In terms of COI, the most abundant order is Perciformes, followed by Cypriniformes and Siluriformes (Table 1). Similarly, among the 18S rRNA sequences, the most frequently sequenced order is also Perciformes, followed by Syngnathiformes and Siluriformes (Table 1).

**Table 1.** The number of COI and 18S rRNA sequences contained in the CoSFISH database

Order	Number of 18S rRNA sequences	Number of COI sequences
Acanthuriformes	22	320
Acipenseriformes	24	28
Acropomatiformes	2	130
Albuliformes	1	11
Alepocephaliformes	0	56
Amiiformes	1	1
Amphioxiformes	3	8
Anabantiformes	18	203
Anguilliformes	23	532
Argentiniformes	2	49
Ateleopodiformes	0	5
Atheriniformes	18	199
Aulopiformes	3	249
Batrachoidiformes	3	33
Beloniformes	12	181
Beryciformes	3	48
Blenniiformes	10	840
Caproiformes	0	16
Carangiformes	12	186
Carcharhiniformes	8	200
Centrarchiformes	29	172
Ceratodontiformes	3	6
Chaetodontiformes	4	192
Characiformes	52	1595
Chimaeriformes	1	38
Cichliformes	56	641
Clupeiformes	19	414
Coelacanthiformes	1	1
Cypriniformes	56	2090
Cyprinodontiformes	19	540
Echinorhiniformes	1	2
Elopiformes	2	9
Ephippiformes	0	11
Esociformes	1	15
Gadiformes	12	250
Galaxiiformes	19	31
Gerreiformes	1	108
Gobiiformes	38	1584
Gonorynchiformes	2	6
Gymnotiformes	2	164
Heterodontiformes	0	4
Hexanchiformes	1	4
Hiodontiformes	1	2
Holocentriformes	3	79
Istiophoriformes	2	50
Kurtiformes	4	512
Labriformes	13	541
Lamniformes	6	15
Lampriformes	1	18
Lepidogalaxiiformes	1	1
Lobotiformes	1	2
Lophiiformes	0	138
Lutjaniformes	7	293
Moroniformes	5	8
Mugiliformes	10	288
Myctophiformes	1	197
Myliobatiformes	6	177
Myxiniformes	0	35
Notacanthiformes	0	22
Ophidiiformes	9	190
Orectolobiformes	7	23
Osmeriformes	9	57
Osteoglossiformes	7	117
Ovalentaria	4	187
Pempheriformes	0	6
Perciformes	142	2520
Percopsiformes	0	7
Petromyzontiformes	3	38
Pleuronectiformes	43	470
Polymixiiformes	0	6
Polypteriformes	2	6
Priacanthiformes	1	37
Rajiformes	3	143
Rhinopristiformes	1	25
Salmoniformes	20	140
Scombriformes	22	254
Semionotiformes	2	8
Siluriformes	84	1853
Spariformes	31	391
Squaliformes	5	125
Squatiniformes	1	24
Stomiiformes	0	194
Stylephoriformes	0	1
Synbranchiformes	2	77
Syngnathiformes	94	571
Tetraodontiformes	28	341
Torpediniformes	1	30
Trachichthyiformes	1	24
Uranoscopiformes	6	97
Zeiformes	1	23
Total	1074	21 535

### Database homepage

**Figure 1.**
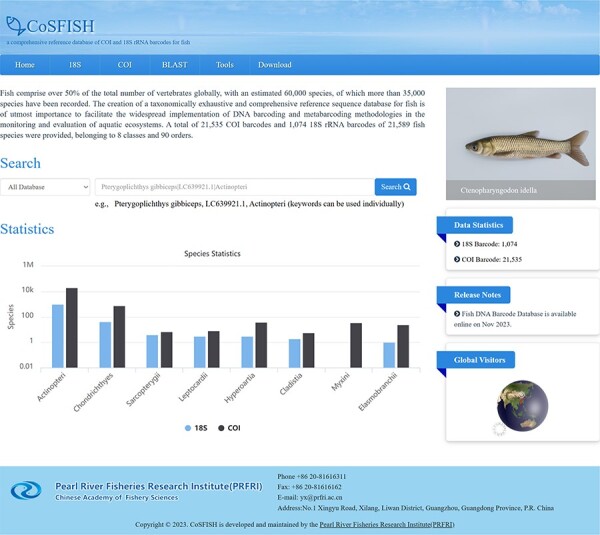
Home page of the CoSFISH database.

The CoSFISH database provides a user-friendly web interface to access and analyze the data. The homepage of the database is divided into two sections: a navigation bar at the top and the main information below the navigation bar. The navigation bar consists of six core functions: ‘Home’, ‘18S’, ‘COI’, ‘BLAST’, ‘Tools’ and ‘Download’. Above the navigation bar is the name and logo of the database. The main information includes the introduction of the database, some representative fish pictures, quick search box, data statistics, release notes and global visitors (Figure 1).

### Search and browse

Search functions can be reached by several different approaches, such as quick search, advanced search and BLAST. The home page search box provides users with the ability to perform quick searches by taxonomic level, accession number or species name. Subsequently, CoSFISH generates a list of search results that correspond to the provided input information. Each result in the list includes a hyperlink to the detailed page of the sequence and respective species (Figure 2A). Each sequence page includes basic information (Figure 2B), geographic distribution description (Figure 2C), references of each sequence (Figure 2D) and taxonomic information (Figure 2E) of the corresponding species. Additionally, we also provide the source link of FishBase and NCBI of each sequence and species, shown in Figure 2B.

**Figure 2.**
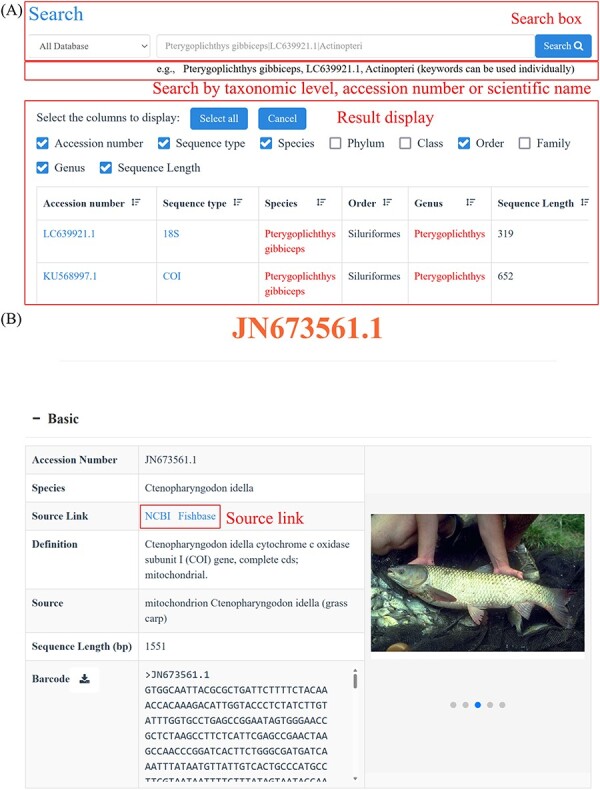
Quick search page (A) and the detail page of each sequence (B–E). (**A**) Example of the quick search result. (**B**) Basic information of each sequence and its corresponding species. (**C**) Geographic distribution information of the species corresponding to the sequence. (**D**) References of the sequence. (**E**) Taxonomic information of the species corresponding to the sequence.

**Figure 2.**
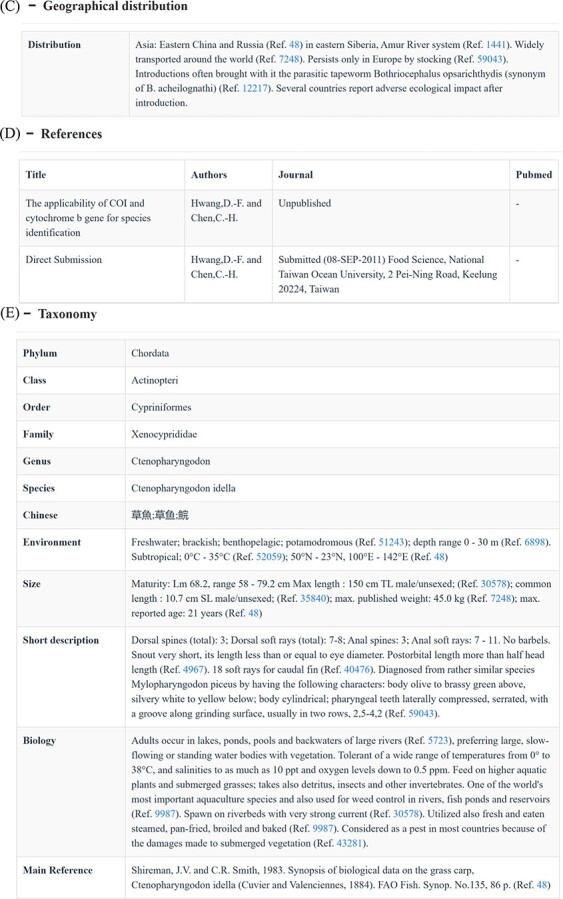
(Continued)

Through the ‘18S’ and ‘COI’ function in the navigation bar, users can realize the advanced search. Each of the ‘18S’ and ‘COI’ page includes two parts: browse and taxonomy. Users can access all the ‘18S’ and ‘COI’ data through the browse function, which enables users to selectively display the desired information, like Accession number, Species, Phylum, Class, Order, Family, Genus and Length (Figure 3A). Simultaneously, the function of taxonomy offers a more flexible way to find specific information. Users can click the taxonomy to choose the interested Phylum, Class, Order, Family, Genus and Species, effectively obtaining the target information. Particularly, for each taxonomic level, the corresponding sequence number is displayed on the right, as well as the corresponding result page (Figure 3B). Besides, we have incorporated the BLAST function into our database to facilitate the comparison of all DNA sequences, resulting in a descending similarity result list. Users can establish parameters to regulate the level of search sensitivity and determine the format of the search results (Figure 3C).

**Figure 3.**
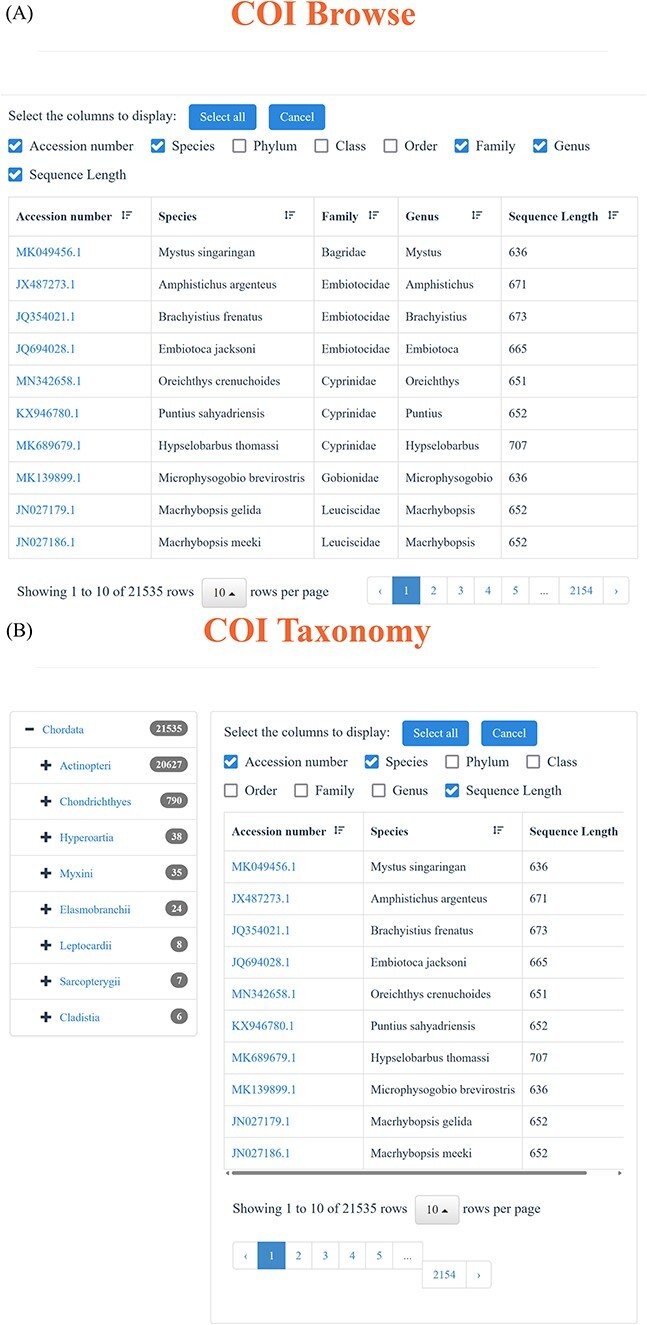
Advanced search function. (**A**) Using ‘browse’ function under ‘18S’ and ‘COI’ function in the navigation bar to view the available sequences. (**B**) Through ‘taxonomy’ function under ‘18S’ and ‘COI’ function in the navigation bar to find the desired sequences. (**C**) BLAST page and an example of the BLAST search result.

**Figure 3.**
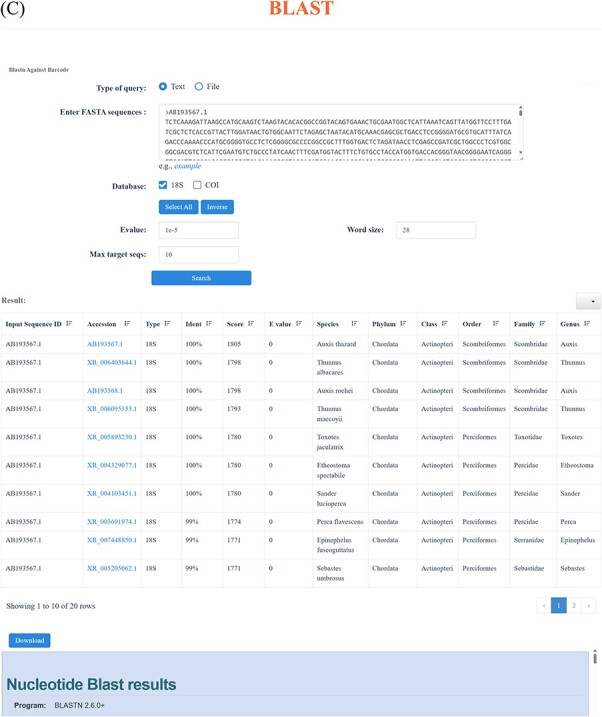
(Continued)

### Online analysis tools

Several online analysis tools are available in the CoSFISH database, including BLAST (17), Muscle (18), Genewise (19), Lastz (20) and Primer3 (21). Given the complexity and time-consuming process of phylogenetic analysis, we also provide a link to CIPRES (22), a professional online platform dedicated to conducting phylogenetic analyses. Users have the option to either upload their own data in FASTA or TXT format or utilize the data available in the CoSFISH database. These tools can help users compare, align and analyze DNA sequences and design primers as well. Upon completion of the analysis, an automated result page will be presented, along with downloadable result files for users to access.

### Download

**Figure 4.**
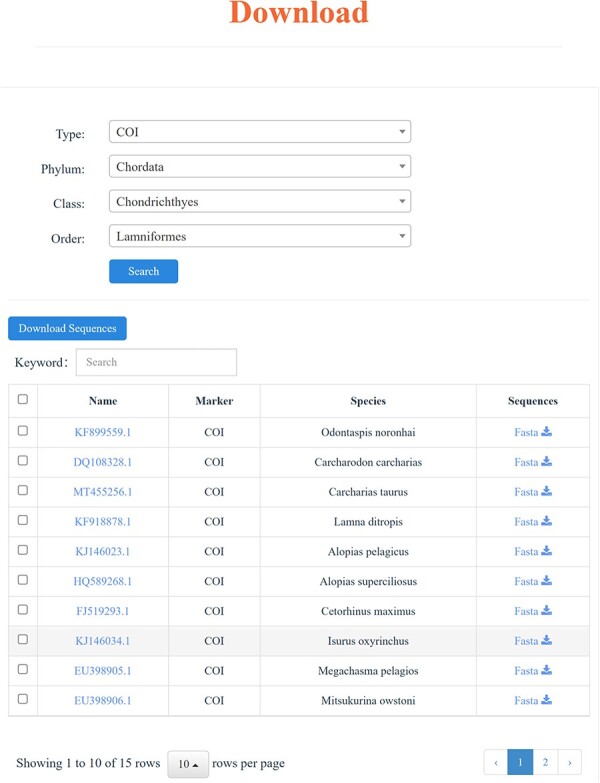
Download page and an example of the download result.

All the COI and 18S rRNA sequences contained within the CoSFISH database are readily accessible and freely downloadable in FASTA or TXT format. Specifically, users can selectively download DNA sequences by marker type, Phylum, Class and Order, based on their academic objectives (Figure 4). For analysis results obtained through online tools, we also offer the option to download the result files in TXT, Newick or SVG formats. In addition, MD5 (Message Digest Algorithm 5) checksums are provided to verify the integrity of the downloaded data.

## Conclusion

To provide a taxonomically complete and comprehensive reference sequence database for fish, we have developed the CoSFISH database, which serves as a valuable resource for the exhaustive study of fish diversity, phylogenetics and biological evolution. CoSFISH stores 21 535 COI sequences and 1074 18S rRNA sequences of 21 589 fish species and provides online analysis tools to compare sequence similarity, conduct phylogenetic analysis and design primers. We will continuously update the CoSFISH database once COI and 18S rRNA sequences of new fish species are released. In addition, we will incorporate a wider range of bioinformatic analysis tools into CoSFISH to enhance its functionality, facilitating the creation of a more comprehensive database for sharing, integrating and utilizing fish genetic resources.

## Data Availability

All data in this study can be accessed through the web server at http://210.22.121.250:8888/CoSFISH/home/indexPage.
